# Endoplasmic reticulum protein 29 (ERp29) confers radioresistance through the DNA repair gene, O^6^-methylguanine DNA-methyltransferase, in breast cancer cells

**DOI:** 10.1038/srep14723

**Published:** 2015-09-30

**Authors:** Shaohua Chen, Yu Zhang, Daohai Zhang

**Affiliations:** 1 Department of Gastroenterology, The First Affiliated Hospital, College of Medicine, Zhejiang University, Hangzhou, China; 2 Department of Oncology, Zhejiang Hospital, Hangzhou, China; 3 Caner Research Group, The Canberra Hospital, ANU Medical School, Australia National University, ACT 2605, Australia

## Abstract

Resistance of cancer cells to radiotherapy is a major clinical problem in cancer treatment. Therefore, understanding the molecular basis of cellular resistance to radiotherapy and identification of novel targets are essential for improving treatment efficacy for cancer patients. Our previous studies have demonstrated a significant role of ERp29 in breast cancer cell survival against doxorubicin-induced genotoxic stress. We here reported that ERp29 expression in the triple negative MDA-MB-231 breast cancer cells significantly increased cell survival against ionizing radiation. Methylation PCR array analysis identified that ERp29 expression increased promoter hypomethylation of the DNA repair gene, O^6^-methylguanine DNA-methyltransferase (*MGMT)*, by downregulating DNA methyltransferase 1. Knockdown of MGMT in the ERp29-transfected cancer cells increased radiosensitivity, leading to a decreased post-irradiation survival. In addition, radiation treatment in the MGMT-knockdown cells elevated phosphorylation of γ-H2AX and cleavage of caspase 3, indicating that depletion of MGMT facilitates DNA double strands breaks and increases cell apoptosis. Hence, our studies prove a novel function of ERp29\MGMT in cancer cell survival against radiation. Targeting ERp29\MGMT axis may be useful for providing better treatment efficacy in combination with radiotherapy in breast cancer.

The endoplasmic reticulum (ER) is a cellular compartment that physiologically involves in protein synthesis and maturation. Perturbation of ER homeostasis results in accumulation of misfolded or unfolded proteins leading to ER stress. Under ER stress, ER chaperones are up-regulated to facilitate cell survival and attenuate apoptotic stimuli. Of the ER chaperones, protein disulfide isomerase (PDI)-like proteins are characterized by the presence of a thioredoxin domain and function as oxido-reductases, isomerases and chaperones^1^. The ER chaperons such as PDI, ERp72 and ERp57 contain an active-site double-cysteine motif and thus have oxido-reductase activity. However, ERp29 does not have this motif and is a redox-inactive PDI-like protein^2^. The structural variation may implicate its different functions in cells, particularly in cancer cells.

ERp29 is considered to be key player in both viral unfolding and secretion^3^,^4^,^5^. Recent studies have demonstrated that ERp29 is involved in intercellular communication by stabilizing the monomeric gap junction protein connexin43^6^ and trafficking of cystic fibrosis transmembrane conductance regulator to the plasma membrane in cystic fibrosis (CF) and non-CF epithelial cells^7^. Furthermore, MDA-MB-231 breast cancer cells express high level of breast cancer stem cell marker CD44 at the cell membrane and has been used as a triple negative and stem-like breast cancer cell model^8^. Overexpression of ERp29 in this type of cells up-regulated the expression of adherens/tight junctions proteins (*e.g*., E-cadherin and ZO-1) and polarity proteins (*e.g*., Par3 and Scribble) to establish epithelial cell integrity and polarity^9^. Recently, it has been reported that ERp29 was highly induced in cells treated with doxorubicin (DOX) and radiation, and the enhanced expression of ERp29 was responsible for cell survival against genotoxic stress^10^,^11^,^12^, Mechanistic studies indicated a pivotal role for ERp29 in cell survival that involved in, at least in part, the enhanced expression of XBP1/p58^IPK^^13^ and Hsp27^12^. DOX is one of the most effective anticancer drugs by exerting its cytotoxic effect through the generation of DNA double-strand breaks (DSBs), formation of oxygen free radicals and intercalation of DOX-DNA adducts that prevent DNA replication^14^,^15^. Besides, radiation can also lead to the formation of DNA-DSBs that are considered the most lethal form of DNA damage^16^. Therefore, the ERp29-induced resistance to DOX and radiation is largely attributable to the capacity of cells to prevent DNA-DSBs formation and/or to repair the damaged DNA.

DNA repair in cells is initiated through nucleotide excision repair, base excision repair, non-homologous end joining, homologous recombinational repair, and mismatch repair^16^. To overcome the DNA-damage induced cell apoptosis, a variety of DNA repair-related genes are activated and exert their repairing function. These DNA repair-related genes include those participating in DNA–DNA crosslink repair, DNA–protein crosslink repair, direct reversal repair, DNA damage signal transduction, and the genes with putative DNA repair functions^16^. Notably, the *O*^6^-methylguanine-DNA methyltransferase (MGMT) has been studied mostly as a DNA repair enzyme *via* rapidly reversing alkylation, including methylation, at the *O*^6^ position of guanine by transferring the alkyl group to the active site of the enzyme^17^. MGMT repairs the mutagenic and cytotoxic interstrand DNA crosslinks resulting after alkylating agent attack and is associated with resistance to alkylating agents in cancer therapy. In addition to DNA repair function, MGMT plays a role in integrating DNA damage/repair related signals that functionally regulate replication, cell cycle progression and genomic stability^18^,^19^. Thus, suppressing MGMT has been considered as an alternative to enhance alkylating/cytotoxic drug treatment^20^,^21^.

This study was undertaken to investigate if ERp29 expression confers to radioresistance and the mechanisms involved using MDA-MB-231 as a cell model. We showed that over-expression of ERp29 significantly increased post-irradiation survival of MDA-MB-231 cells via epigenetic regulation of MGMT. Depletion of the ERp29-induced MGMT by siRNA in MDA-MB-231 cells and MCF-7 cells increased radiosensitivity, activated DNA-DSBs-related H2AX phosphorylation and induced the expression of cleaved caspase 3 and apoptosis. Our studies highlight a critical role of ERp29\MGMT axis in DNA repair and cell survival against irradiation.

## Results

### ERp29 expression increases post-irradiation survival of MDA-MB-231 cells

**Figure 1:**
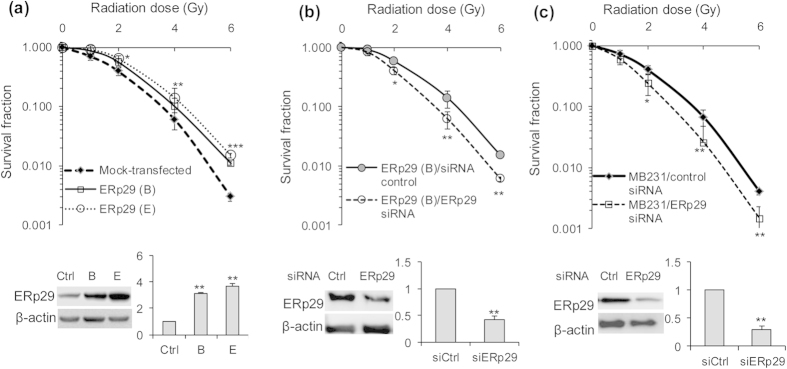
ERp29 regulates post-irradiation survival of MDA-MB-231 cells. (**a**) ERp29 overexpression increased post-irradiation survival rate. ERp29 expressing construct was transfected into MB-231 cells and two stable clones (clone B and E) showing high expression of ERp29 were selected for radiation treatment. (**b**) Repression of exogenously expressed ERp29 by siRNA in the ERp29-transfected MDA-MB-231 cells (clone B) attenuated the post-irradiation survival rate. (**c**) ERp29 knockdown by siRNA in parent MB-231 cells reduced post-irradiation survival rate. ERp29-transfected or knockdown cells (48 hours of treatment with siRNA #1) were seeded on six-well plates and irradiated with the indicated dose of radiation. After 10 days incubation at 37 °C, colonies with >50 cells per colony were counted. The survival fraction of irradiated cells was normalized to the plating efficiency of non-irradiated control cells. The level of ER29 in ERp29-transfected cells (**a**) siRNA-treated, ERp29-overexpressed clone B cells (**b**) and siRNA-treated parental MDA-MB-231 cells (**c**) was examined by Western blot. Data represent the mean ± SD of three independent experiments.*p < 0.05, **p < 0.01, ***p < 0.001, relative to controls at the indicated dose. The level of β-actin was used as a loading control.

To understand whether ERp29 expression confers to radioresistance in MDA-MB-231 breast cancer cells, the ERp29-transfected cells (MB-231/ERp29) and mock-transfected (MB-231/vector) control cells were exposed to ionizing radiation (0–6 Gy) and the post-irradiation survival of cells was analysed by clonogenic assay. The radiation dose, D37, which is required to reduce cell survival to 37%, was determined to evaluate radiosensitivity. As indicated in Fig. 1, ERp29 expression in MDA-MB-231 cells (clone B and E) resulted in highly increase of D37 compared to the mock-transfected control cells (clone B: 2.59 ± 0.15 Gy, clone E: 2.84 ± 0.13 Gy *vs.* control: 2.11 ± 0.14 Gy, p < 0.01).

To further substantiate the ERp29’s role in radioresistance in this cell type, the exogenously expressed ERp29 was depleted by treatment with ERp29 siRNA (Suppl. Fig. 1A, Fig. 1B). In line with the reduction of ERp29 in clone B cells, the enhanced post-irradiation survival rate (D37) in clone B cells was attenuated from 2.68 ± 0.12 to 2.15 ± 0.08 (p < 0.05, Fig. 1b). In addition, the endogenous ERp29 in the parent MDA-MB-231 cells was also reduced by siRNA (Suppl. Fig. 1B; Fig. 1 C). Repression of ERp29 led to a decrease of post-irradiation survival (D37) to 1.68 ± 0.10 Gy, compared to the cells pre-treated with non-targeted siRNA (2.15 ± 0.11 Gy, p < 0.05, Fig. 1c). Therefore, ERp29 exerts a radioresistant function in MDA-MB-231 cells.

### ERp29 expression up-regulates MGMT expression *via* promoter hypomethylation in MDA-MB-231 cells

**Figure 2:**
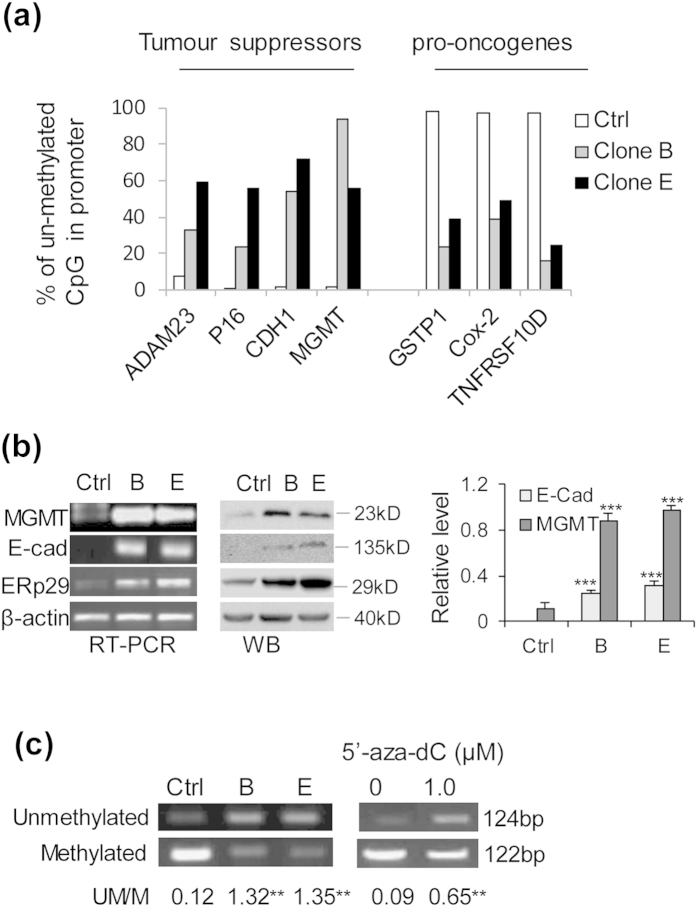
ERp29 modulates the expression of tumour suppressors and pro-oncogenes by epigenetic regulation. (**a**) ERp29 expression promoted/inhibited promoter demethylation of tumour suppressors/pro-oncogenes identified by Methylation PCR arrays. (**b**) Tumour suppressor genes *CDH1* and *MGMT* were transcriptionally activated by ERp29. The mRNA and protein expressions were examined by RT-PCR and Western blot. (**c**) MS-PCR analysis for *MGMT* promoter methylation/demethylation. Note that the ratio of demethylation/methylation was highly increased in the ERp29-transfected cells (clone B and E). Cells treated with 5′-aza-dC was used as a positive control for demethylation. Genomic DNA was extracted and converted with sodium bisulfite. MS-PCR was performed as described in “Materials and Methods”. **p < 0.01 versus control.

Our previous studies showed that overexpression of ERp29 significantly increased the expression of tumour suppressors, such as E-cadherin (CDH1), at both mRNA and protein levels^22^. Given that expression of this tumour suppressor has been found to be regulated by epigenetic mechanism^23^,^24^, the role of ERp29 in epigenetic regulation was investigated using a Methyl-Profiler™ DNA Methylation PCR Array in mock-transfected control cells and MB-231/ERp29 cells. Interestingly, we identified that over-expression of ERp29 remarkably enhanced promoter demethylation of tumour suppressor genes including *p15, CDH1* and *MGMT* (Fig. 2a). For instance, the percentage of hypomethylation of *CDH1* and *MGMT* promoters was increased from approximately 2% in mock-transfected control cells to 55–70% and 55–95%, respectively, in MB-231/ERp29 cells (clone B and E), resulting in increased mRNA and protein expression of CDH1 and MGMT (Fig. 2b). Moreover, it was also found ERp29 overexpression in MDA-MB-231 cells decreased promoter demethylation in several pro-oncogenes (*e.g., Cox-2,* Fig. 2a).

To further verify the regulatory role of ERp29 in methylation/demethylation, MS-PCR was used to analyse the methylation status of the promoter region (*e.g*., 3^rd^ CpG islands) of *MGMT* in these cell models. Compared to mock-transfected control cells, the MB-231/ERp29 cells showed a significant reduction of methylation and increase of demethylation of *MGMT* promoter, similar to those observed in MDA-MB-231 cells treated with 5′-aza-dC (Fig. 2c). These results suggest that ERp29 expression is able to re-activate *MGMT* transcription and expression by epigenetic regulation in MDA-MB-231 cells.

### ERp29 regulates MGMT promoter methylation via DNMT1 in MDA-MB-231 cells

**Figure 3:**
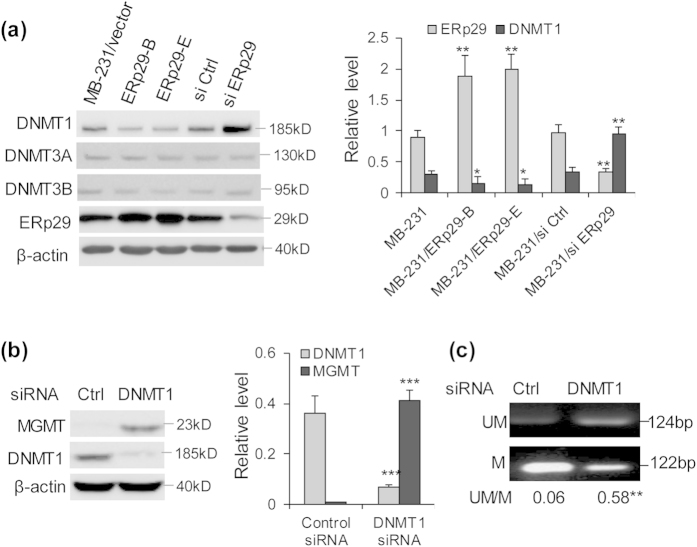
ERp29 expression reduces DNMT1 to increase MGMT promoter demethylation in MDA-MB-231 cells. (**a**) ERp29 expression decreased the level of DNMT1 whereas ERp29 knockdown upregulated the expression of DNMT1. The expression of DNMT3A or 3B was not markedly affected by ERp29. *p < 0.05, **p < 0.01, relative mock-transfected control or controL siRNA. (**b**) Reduction of DNMT1 by siRNA upregulated MGMT expression in MDA-MB-231 cells. MDA-MB-231 cells were transiently transfected with control siRNA or *DNMT1* siRNA (#1) for 48hours and the expression of DNMT1 and MGMT was examined. (**c**) *MGMT* promoter methylation/demethylation. Genomic DNA was extracted from the MDA-MB-231 cells transfected with control siRNA or *DNMT1* siRNA and the MS-PCR was done as described in “Materials and Methods”. **p < 0.01, ***p < 0.001, versus control.

Since DNA methyltransferase is responsible for increase of DNA methylation, the expression of DNMT1, DNMT3A and DNMT3B was analysed in mock-transfected control cells and MB-231/ERp29 cells. As indicated in Fig. 3a, relative to control cells, ERp29 overexpression in MDA-MB-231 cells significantly inhibited the expression of DNMT1, rather than the expression of DNMT3A or 3B. The role of DNMT1 in epigenetic regulation of MGMT expression was further supported by the fact that DNMT1 knockdown by siRNA in MDA-MB-231 cells (Suppl. Fig. 1C) led to an increase of MGMT expression compared to the cells treated with non-targeted control siRNA (Fig. 3b). MS-PCR analysis showed that DNMT1 knockdown in MDA-MB-231 cells enhanced demethylation and reduced methylation of *MGMT* promoter relative to the cells treated with control siRNA (Fig. 3c). These data indicate a critical role of DNMT1 in ERp29-mediated inhibition of *MGMT* promoter methylation.

### MGMT is a downstream target regulated by ERp29

**Figure 4:**
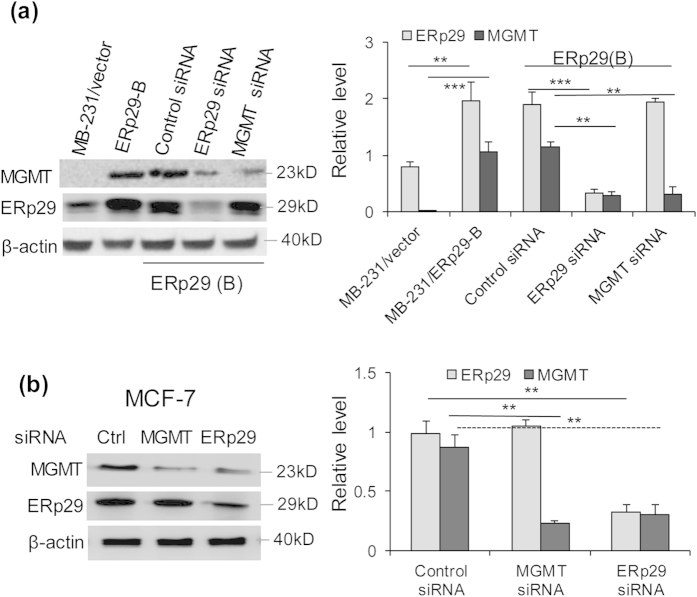
MGMT is a downstream target of ERp29. ERp29-transfected cells (cone B) (**a**) or MCF7 cells (**b**) were treated with *ERp29* siRNA (#1) or *MGMT* siRNA (#3) or control siRNA and the expression of ERp29 and MGMT was analysed. ERp29 knockdown decreased the expression of MGMT whereas MGMT knockdown was unable to decrease the level of ERp29 in both clone B cells (**a**) and MCF-7 cells (**b**). **p < 0.01, ***p < 0.001, relative to control.

To further understand whether MGMT is a downstream target of ERp29, the MB-231/ERp29 cells (clone B) were respectively treated for 48 h with MGMT siRNA, or ERp29 siRNA, or the non-targeted control siRNA. We showed that depletion of ERp29 in MB-231/ERp29 cells reduced the level of MGMT compared to those treated with control siRNA (Fig. 4a). However, depletion of MGMT was unable to affect the level of total ERp29 (endogenously and exogenously expressed) in these cells (Fig. 4a). This is reflected by the fact that the overall ERp29 level in the MGMT siRNA-treated MB-231/ERp29 cells was similar to that expressed in the untreated or control siRNA-treated MB-231/ERp29 cells.

Because MDA-MB-231 parent cells showed weak expression of MGMT as assessed by Western blot (Fig. 2b), knockdown of endogenous MGMT was performed in MCF-7 cells which express high levels of both ERp29 and MGMT. Knockdown efficiency of ERp29 and MGMT by siRNA in MCF-7 cells were assessed (Suppl. Fig. 1D and E). As demonstrated in Fig. 4b, reduction of endogenous ERp29 by siRNA decreased the level of MGMT. Nevertheless, repression of endogenous MGMT by siRNA was unable to reduce ERp29 expression in MCF-7 cells. These results support that MGMT is a downstream target of ERp29.

### MGMT mediates ERp29-induced radioresistance in MDA-MB-231 cells

**Figure 5:**
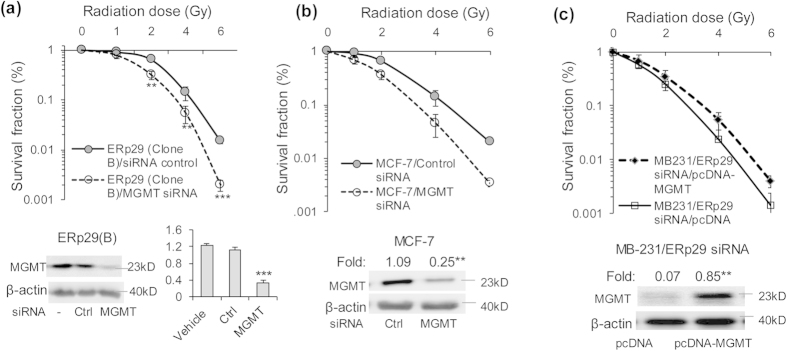
MGMT mediates ERp29-induced post-irradiation survival rate. and facilitates DNA damage in ERp29-transfected MDA-MB-231 cells. (**a**) Repression of MGMT by siRNA (#3) reduced the ERp29-enhanced post-irradiation survival rate. Cells were treated with control or *MGMT* siRNA (#3) for 48 hours and then exposed to irradiation at the indicated doses. Expression of MGMT was efficiently repressed by siRNA in the ERp29-overexpressed clone B cells. (**b**) Depletion of endogenous MGMT by siRNA in MCF-7 cells sensitized to radiation treatment. MCF-7 cells were treated with control or *MGMT* siRNA (#3) for 48 hours and then exposed to irradiation at the indicated doses. The expression of MGMT in MCF-7 cells was efficiently reduced by siRNA. (**c**) Re-expression of MGMT in the MB-231/ERp29 siRNA cells restores radioresistance. Cells were transfected with pcDNA-MGMT or pcDNA for 24 hours and exposed to radiation treatment. MGMT expressed was examined by immunoblot. Post-radiation survival rate was assessed by clonogenic assay as described in Fig. 1

We showed that ERp29 overexpression resulted in radioresistance in MDA-MB-231 cells (Fig. 1). To understand whether the upregulated MGMT by ERp29 is involved in radioresistance, the MB-231/ERp29 cells (clone B) were treated for 48 hours with MGMT siRNA to reduce MGMT expression or with control siRNA. These cells were then irradiated (0–6 Gy) and the post-irradiation survival assessed using the clonogenic assay. Relative to MB-231/ERp29 cells treated with control siRNA (D37 = 2.72 ± 0.09 Gy), MGMT knockdown in these cells caused a significantly decrease of D37 (1.82 ± 0.13 Gy, p < 0.05) (Fig. 5a). These data indicate that MGMT depletion re-sensitizes to radiation in MB-231/ERp29 cells.

The role of MGMT in protecting cells from radiation-induced cell death was further investigated in MCF-7 cells which showed relative high expression of endogenous MGMT (Fig. 4b). The endogenously expressed MGMT in MCF-7 cells was markedly repressed by treatment with MGMT siRNA for 48 hours (Suppl. Fig. 1D) and this caused a significant reduction of the post-irradiation cell survival rate (D37) from 2.82 ± 0.09 to 2.01 ± 0.11 (p < 0.05, Fig. 5b). These data further suggest MGMT is critical for radioresistance in MCF-7cancer cells.

To understand whether re-expression of MGMT can restore radioresistance in the ERp29 knockdown-induced radiosensitive cells, pcDNA-MGMT and pcDNA control vectors were respectively transfected into MB-231/ERp29 siRNA cells. As indicated in Fig. 5c, MGMT was highly expressed after 24hours post-transfection compared to the pcDNA transfected cells. Clonogenic assay showed that MGMT re-expression led to an increase of D37 compared to control cells (2.02 ± 0.16 Gy *vs.* 1.65 ± 0.21 Gy, p < 0.05). This rescue experiment resulted in >95% reversion to radioresistance in the MB-231/ERp29 siRNA cells, comparing to the D37 values in mock-transfected MB-231 cells (D37: 2.11 ± 0.14 Gy, Fig. 1a) and non-target siRNA-treated MB-231 cells (D37: 2.15 ± 0.11 Gy, Fig. 1c). Taken together, the ERp29-induced MGMT is a key molecule promoting ERp29-mediated radioresistance in breast cancer cells.

### Knockdown of MGMT reduces DNA repair capacity and enhances DNA damage after irradiation

**Figure 6:**
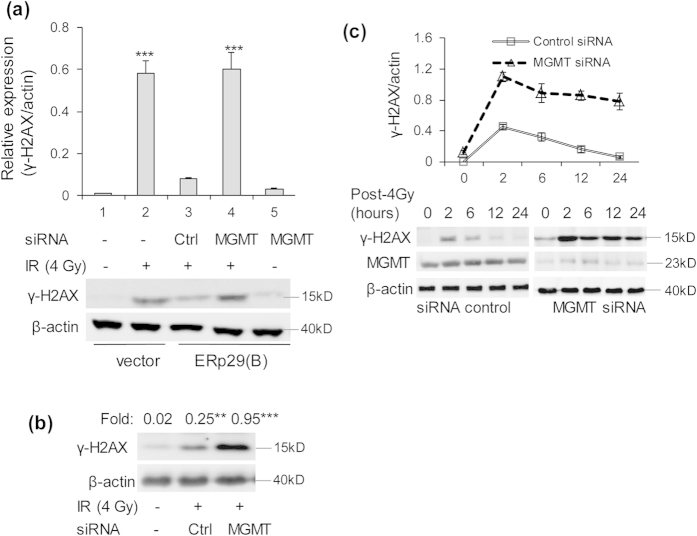
MGMT repression facilitates DNA damage in ERp29-transfected MDA-MB-231 cells and MCF-7 cells. (**a**) Reduction of MGMT in ERp29-overexpressed cells increased irradiation-induced expression of γ–H2AX. Note that irradiation induces significant increase of γ–H2AX in the mock-transfected MDA-MB-231 cells (column 2 *vs*. 1). Depletion of MGMT alone in the ERp29-overexpressed clone B cells only slightly increased the expression of H2AX (column 5 *vs.* 1). However, combination treatment (column 4) of irradiation/*MGMT* siRNA in these cells led to a significant induction of γ–H2AX relative to the cells treated with MGMT siRNA (column 5) or with the irradiation/control siRNA (column 3). The level of γ–H2AX was examined after 12 hours of post-irradiation. (**b**) Irradiation treatment significantly increased the expression of γ–H2AX in MGMT-knockdown MCF-7 cells. MCF-7 cells were transfected with *MGMT* siRNA or control siRNA for 48 hours and then irradiated with 4 Gy. The expression of γ–H2AX was assayed after 12 hours of post-irradiation. (**c**) MGMT depletion induced high expression of γ–H2AX and reduced double strand DNA breaks repair after irradiation. MB-231/ERp29 (clone B) cells were treated with *MGMT* siRNA or control siRNA for 48 hours, irradiated with 4 Gy and incubated for 2, 6, 12 and 24 hours. Phosphorylation of H2AX was examined by Western blot. **p < 0.01, ***p < 0.001, relative to control at the indicated doses/time.

Phosphorylation of H2AX is a marker for the cellular response to DNA-DSBs^25^. Upon DNA damage in cells, histone H2AX is phosphorylated on serine 139 to generate γ-H2AX^26^. Hence, γ-H2AX expression after ionizing radiation reflects the formation of double strands DNA breaks and DNA repair capacity. To investigate the effect of the upregulated MGMT by ERp29 on the expression of γ-H2AX, the MB-231/ERp29 cells (clone B) were transfected with non-target control siRNA or MGMT siRNA for 48h. These cells, together with vector-transfected control cells, were mock-irradiated or irradiated with 4 Gy. After 12 hours of incubation, the expression of γ-H2AX was assessed. As indicated in Fig. 6a, irradiation treatment significantly increased the expression of γ-H2AX in vector-transfected control cells relative to the mock-irradiation treatment (column 2 *vs.* 1). However, compared to the irradiation-treated vector-transfected control cells, irradiation treatment only moderately increased the expression of γ-H2AX in MB-231/ERp29 cells (column 3 *vs.* 2), indicating a protective role of ERp29 from the irradiation treatment. Nevertheless, knockdown of the ERp29-upregulated MGMT by siRNA markedly increased radiation-induced expression of γ-H2AX, relative to MB-231/ERp29 cells pre-treated with control siRNA (column 4 *vs.* 3; Fig. 6a). Similarly, knockdown of the endogenously expressed MGMT in MCF-7 cells led to significant increase of γ-H2AX expression, compared to the cells treated with control siRNA after radiation treatment (Fig. 6b).

To understand the effect of MGMT depletion on the kinetics of γ-H2AX after irradiation, the MB-231/ERp29 cells were treated with non-target control siRNA or MGMT siRNA for 48 h and then irradiated at 4 Gy. The level of γ-H2AX was analysed at 0, 2, 12 and 24 hours post-irradiation (Fig. 6c). For the MB-231/ERp29 cells treated with control siRNA, the level of γ-H2AX was rapidly increased within 2 hours and then decreased after irradiation. In contrast, MGMT knockdown in MB-231/ERp29 cells resulted in a sustained high expression and slow reduction of γ-H2AX within 24 hours. These results suggest that loss of MGMT by siRNA leads to increased double strand DNA breaks and impairment of DNA repair capacity in these cells.

### Knockdown of MGMT enhances cell apoptosis in the ERp29-transfected MDA-MB-231 cells

**Figure 7:**
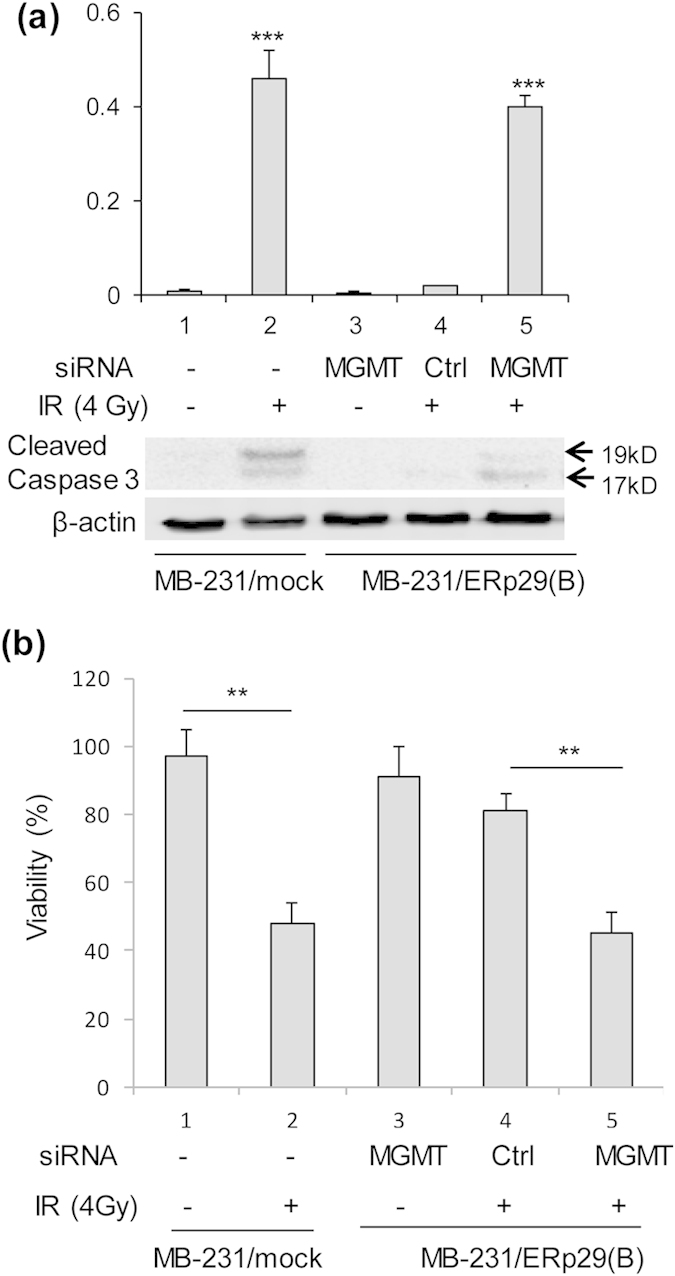
MGMT depletion by siRNA stimulates radiation-induced cell apoptosis in ERp29-transfected MDA-MB-231 cells. (**a**) Reduction of MGMT in ERp29-overexpressed clone B cells increased irradiation-induced expression of cleaved caspase 3 compared to the control siRNA-treated cells. Expression of cleaved caspase 3 was highly induced by irradiation in the vector-transfected MDA-MB-231 cells (column 2 *vs.* 1). In the ERp29-overespressed clone B cells, depletion of MGMT alone did not activate expression of cleaved caspase 3 (column 3 *vs.* 1). However, these cells treated with *MGMT* siRNA (#3) markedly increased the expression of cleaved caspase 3 after irradiation (column 5 *vs.* 4). (**b**) Cell viability. Irradiation treatment caused significant reduction of cell viability in vector-transfected MDA-MB-231 cells (column 2 *vs.* 1). Combinatory treatment of irradiation/*MGMT* siRNA (column 5) in the ERp29-overexpressed clone B cells significantly reduced cell viability compared to the cells treated with *MGMT* siRNA alone (column 3) or the irradiation/control siRNA (column 4). **p < 0.01, ***p < 0.001, relative to control.

To examine whether the ERp29-induced activation of MGMT is responsible for antagonizing radiation-induced apoptosis, the MB-231/ERp29 cells were treated with *MGMT* siRNA or control siRNA for 48 hours and exposed to radiation treatment (4 Gy). After 48 hours post-incubation, these cells were used for examining the expression of cleaved caspase 3 and cell viability. In parallel, the vector-transfected control cells were also exposed to radiation treatment (4 Gy). As shown in Fig. 7a, radiation treatment significantly increased the expression of cleaved caspase-3 (column 2 *vs.* 1) in the vector-transfected control cells. Interestingly, MGMT depletion alone in MB-231/ERp29 cells could not activate the expression of cleaved caspase 3 (column 3). However, compared to MB-231/ERp29 cells pre-treated with control siRNA, knockdown of the ERp29-induced MGMT significantly increased the expression of cleaved caspase-3 after exposure to irradiation (column 5 *vs.* 4). Hence, MGMT depletion facilitates the radiation-induced caspase activation in MB-231/ERp29 cells.

To further establish the role of MGMT in cell survival after irradiation, these cells were applied for viability analysis using the MTT assay. Our data showed that irradiation caused >50% reduction of viable cells in the vector-transfected control cells (column 2 *vs.* 1; Fig. 7b). In MB-231/ERp29 cells, MGMT depletion alone or treated with control siRNA/radiation was unable to significantly decrease the cell viability compared to the untreated vector-transfected control cells (column 3 *vs.* 1, and 4 *vs.* 1; Fig. 7b). However, when the MB-231/ERp29 cells were exposed to a combinatory treatment with MGMT siRNA and radiation, the cell viability was remarkably reduced by >40% compared to those treated with control siRNA/radiation (column 5 *vs.* 4; Fig. 7b). Therefore, depletion of MGMT in the MB-231/ERp29 cells significantly increases radiation-induced cell death.

## Discussion

Previous studies have demonstrated a significant role of ERp29 in the resistance to DOX in breast cancer cells^12^. The present work has further established a critical function of ERp29 in radioresistance through a mechanism involved in epigenetic regulation of the DNA repair gene, *MGMT*. Our data suggest that the ERp29\MGMT axis could be a potential target for the treatment of radioresistant cancer cells.

Radiotherapy is a typical approach for cancer treatment. However, some cancer cells develop resistant capacity against irradiation by various mechanisms, such as increased expression of anti-apoptotic proteins and increased ability of DNA repair^27^. Because cancer cell resistance to radiation is a major hurdle of radiotherapy, understanding the molecular mechanisms of radioresistance and identifying the potential targets to enhance radiosensitivity are fundamental to improve the efficacy of radiotherapy. It was reported that ERp29 knockdown attenuated radioresistance and enhanced cell apoptosis in nasopharyngeal cancer cells^11^. In support of this, we showed that ERp29 expression significantly increased the post-irradiation survival and depletion of ERp29 sensitized to radiation, leading to reduced post-irradiation survival in the basal-like MDA-MB-231 cells (Fig. 1). In addition, previous studies indicated that ERp29 expression conferred resistance to DOX, an agent used for killing cancer cells by causing DNA damage and cell apoptosis^28^, in cancer cells^12^. The current studies further provide evidence to support that ERp29 plays a protective role for DNA integrity from the genotoxic stress induced by DOX or irradiation.

In this study, we investigate the molecular base underlying ERp29-mediated radioresistance in MDA-MB-231 breast cancer cells. Using the methylation PCR array, we have demonstrated that overexpression of ERp29 resulted in hypomethylation of CpG islands in tumour suppressor genes such as *CDH1, p16* and *MGMT*, thereby leading to upregulation of these genes. Further studies revealed that ERp29 expression reduced the expression of DNMT1, a key enzyme causing DNA methylation. To our knowledge, this is for the first time to report a novel function of ERp29 in epigenetic regulation in cancer cells, although the mechanisms of action remain to be fully elucidated. Epigenetic regulation is a complex process involves DNA methylation and histone modification. DNA methylation at CpG islands is carried out by one of 3 DNMTs (DNMT-1, −3a & −3b)^29^ and is considered to be a very stable epigenetic modification. However, histone modifications are more labile and regulated by histone acetyltransferase and lysine methyltransferases^30^. Generally, modification of histone H3 at lysine residues 9 or 27 (H3K9 or H3K27) is associated with gene silencing, whereas H3K4 methylation correlates with gene activation^31^. Although our current studies demonstrated an involvement of DNMT1 in the reactivation of MGMT by ERp29, other molecular regulation, *e.g*, histone modifications, should not be excluded.

MGMT is an important DNA repair protein by transferring the methyl group from guanine to its own cysteine residue^32^. Loss of MGMT is unable to remove O^6^-methylguanine (O^6^MeG) which induces DNA-DSBs to trigger apoptosis *via* signalling involved in ATR/ATM, Chk1 and p53^21^. As such, MGMT plays a significant role in DNA repair and cell survival. We demonstrated that MGMT is a critical player in ERp29-mediated radioresistance in MDA-MB-231 cells. Our data showed that depletion of MGMT by siRNA in MB-231/ERp29 cells remarkably reduced the post-irradiation survival of cells. This is further supported by the fact that reduction of MGMT led to an enhanced expression of γ-H2AX, an indicator of DNA-DSBs^33^,^34^, in MB-231/ERp29 cells. In line with this, reduction of MGMT also resulted in an increased expression of cleaved caspase 3 and a reduction of cell viability. Our studies confirm that MGMT knockdown reduced the DNA repair capacity and promotes cell apoptosis in MB-231/ERp29 cells after irradiation treatment. Taken together, the ERp29-upregulated MGMT is a key player in preventing radiation-induced DNA damage in MB-231/ERp29 cells.

In summary, our studies demonstrate a novel function of ERp29\MGMT in maintaining DNA integrity and radioresistance in MDA-MB-231 cells. Hence, targeting the ERp29\MGMT could be an effective strategy to overcome resistance to radiotherapy in cancer. To this end, further studies will be undertaken to investigate the radioresistant function of ERp29\MGMT in other cancer cell models and to investigate the association of this ERp29\MGMT axis with radioresistant phenotype in clinical tumour specimens.

## Materials and Methods

### Antibodies and reagents

The following antibodies were used in this study: rabbit anti-human ERp29 from Novus Biologicals (Littleton, CO); mouse anti-human *β*-actin from Sigma-Aldrich (Saint Louis, MO); rabbit anti-phosphorylated histone variant H2A.X (Ser139), rabbit anti-human MGMT, rabbit anti-human E-Cadherin, rabbit anti-human cleaved caspase 3, rabbit anti-human DNMT1, DNMT3A and DNMT3B and mouse anti-human MGMT from Cell Signalling (Beverly, MA). ERp29 Trilencer-27 Human siRNA, MGMT Trilencer-27 Human siRNA and DNMT1 Trilencer-27 Human siRNA kits include three Dicer-Substrate 27-mer duplexes for each gene and were purchased from Origene (Rockville, MD). 5-Aza-2-deoxycytidine (5-aza-dC) was purchased from Sigma-Aldrich.

### Cell Culture

MDA-MB-231 and MCF-7 breast cancer cell lines were purchased from the American Type Culture Collection (ATCC, Manassas, VA). Vector- and ERp29-transfected MDA-MB-231 cells generated as described previously^22^ were maintained in DMEM supplemented with 10% fetal bovine serum (FBS) and G418 (Life Technologies, Grand Island, NY). Cells were cultured at 37 °C in a humidified incubator with 5% CO_2._

### Irradiation and clonogenic assay

Clonogenic cell survival after irradiation treatment was determined using colony formation assay. Briefly, cells were plated in 6-well plates at appropriate dilutions. After 24 h post-incubation, cells were irradiated with single dose of 1, 2, 4, or 6 Gy at 37 °C with a dose rate 1.7 Gy/min using a Gamma Cell 1000 Elite irradiator (Theratronics, Ottawa, Canada) with a ^137^Cs source. Mock-irradiated cells were used as control. The plates were incubated at 37 °C for 10 days to allow colony formation. Colonies were fixed with methanol and stained with crystal violet. Colonies with >50 cells were counted manually. Clonogenic fractions of irradiated cells were normalized to the plating efficiency of non-irradiated control group. The value of radiation dose D37, a dose required to reduce the number of clonogenic cells to 37%, was used to evaluate radiosensitivity.

### Cell viability

Cells were seeded at 3 × 10^3^ cells per well in 96 well plates and incubated at 37 °C in a cell culture incubator. After 15 hours post-incubation, the cells were exposed to irradiation (4 Gy), followed by incubation for 48 hours. The viable cells were examined using the Vybrant® MTT Cell Proliferation Assay Kit (Life Technologies).

### Methyl-Profiler™ DNA Methylation PCRh

Promoter methylation/demethylation was performed using the Methyl-Profiler™ DNA Methylation PCR Array (SuperArray Bioscience Corporation, Frederick, MD). Briefly, 0.5 *μ*g of total genomic DNA was digested with enzyme A and/or B and the PCR reactions were set up according to the provided protocol. PCR was carried out with the 96-well plate format and the ABI 7500 Fast Real-Time PCR System (Applied Bio systems, Singapore). Gene amplification was detected with SYBR, and data analysis was carried out using the ΔCt method (http://www.sabiosciences.com/methylationdataanalysis.php). The “Results” worksheet displays the relative percentage of hypermethylated, intermediately methylated and unmethylated DNA in each target promoter sequence. Data represent a mean of percentage of unmethylation level from triplicate experiments.

### DNA extracthion, bisulfite treatment and methylation specific PCR (MS-PCR)

Genomic DNA was isolated from cells using the QIAamp DNA Mini Kit (Qiagen, Hilden, Germany) according to the manufacturer’s instructions. The final yield and quality of extracted DNA was measured using Nanodrop 2000c (Thermo Scientific, Wilmington, DE). The DNA (2.5 μg) was then converted by sodium bisulfite treatment using as per standard protocol using the EZ DNA Methylation™ kit (Zymo Research Cooperation, Irvine, CA).

MS-PCR for MGMT was performed using primers distinguishing between methylated and demethylated CpG dinucleotide at the 3^rd^ CpG islands in *MGMT* promoter. Methylation of CpG was examined using the forward primer (5′-CGAAT ATACT AAAACA ACCCG CG-3′) and reverse primer (5′-GTATTTTTTCGGGAGCGAGGC-3′) (Genbank accession X61657 (1029–1150)). Demethylation of CpG was examined using the forward primer (5′-CCAAA TATAC TAAAA CAACC CACA-3′) and reverse primer (5′-TGTAT TTTT TTGGG AGTGA GGT-3′) (Genbank accession X61657 (1028–1152)). As a positive control for DNA demethylation, MDA-MB-231 cells were treated with 5-Aza-dC at 0, and 1.0 μM for 24 hours. The DNA extraction, sodium bisulfite treatment and MS-PCR were carried out by a similar procedure. The PCR products were analysed by electrophoresis on 2% agarose gel and visualized after ethidium bromide staining. Data represent the relative ratio of band intensity of unmethylated/methylated PCR products.

### Reverse transcription (RT)-PCR

Total RNA from cells was extracted using NucleoSpin RNA II (Macherey-Nagel GmbH & Co. KG, Germany), and cDNA was synthesized using ImProm-II reverse transcriptase (Promega) as described previously^22^. The genes *CDH1, MGMT* and *ERp29* were amplified by semi-quantitative PCR using their specific primers (*CDH1:* sense, 5′-TGACAA CGCCC CCATAC CAG-3′and antisense, 5′-TTCTCC GCCTC CTTCT TCATC ATA-3; *MGMT*: sense, 5′-AGTGG TTAGG CGGCA GCAGC-3′ and antisense, 5′-CCACG GTCAG TGTGG GTCAG-3′); *ERp29*: sense, 5′-TCTCC TGGGC TTCCT GCTCC TCTC-3′ and antisense, 5′-TATTG CTCGG CCCAC TTCTT CTGA-3′). As an internal control, β-actin was amplified with sense primer (5′-CCTTC CTGGG CATGG AGTCC TG-3′) and antisense primer (5′-GGACA ATGAT CTTGA TCTTC-3′). A 5 μl aliquot of each of the PCR products was detected on a 1.5% agarose gel with ethidium bromide staining.

### Western Blot Analysis

Western blot was carried out as per standard procedure. In all, 30 *μ*g of protein was resolved by SDS-PAGE and transferred onto a PVDF membrane, and then probed with specific antibodies (ERp29, 1:2000; MGMT, 1: 1000; E-cadherin, 1:1000; γ-H2AX, 1:1000; cleaved caspase 3, 1:2000; DNMT1, 1:1000; DNMT3A, 1:1000; DNMT3B, 1:1000, β-actin, 1:2000). Goat-anti-mouse or goat-anti-rabbit IgG horseradish peroxidase (HRP, Upstate Biotechnology, Lake Placid, NY) was used as secondary antibody. Chemiluminescent signals were visualized using SuperSignal™ West Pico Chemiluminescent Substrate (Thermo Scientific) and signal intensity was analysed using GeneTools software (Syngene, Frederick, MD). The level of *β*-actin was used as loading control.

### RNA interference, MGMT vector and transfection

For knockdown of target genes by siRNA, 3 siRNA duplex provided in the kit were assessed to evaluate the efficiency. In brief, cells at 60–80% confluence were transfected with the gene-specific siRNA duplex (*MGMT, ERp29, DNMT1*) or control siRNA at a final concentration of 50 nM using Lipofectamine™ 2000 transfection reagent (Invitrogen) according to the manufacturer’s instructions. After 48 hours, the cells were harvested and the efficiency of gene knockdown was examined by Western blot (Suppl. Fig. 1).

For construction of MGMT expression vector, *MGMT* human cDNA (Origene) were amplified using forward primer 5′-CG*GGATCC*GG CACCATGGAC AAGGATTGTG AAATGAAACG-3′ with *BamH*I; and reverse primer 5′-C*TCTAGA*TCA GTTTCGGC CAGCAGGCGG GGAGC-3′ with *Xba*I. *MGMT* was then subcloned into pCDNA3.1 (Invitrogen™) after digestion with *BamH*I/*Xba*I. For transient transfection, MDA-MB-231/ERp29 siRNA cells incubated in a 6-well plate were transfected with pcDNA-MGMT or pcDNA at 2 μg per well for 24 hours using lipofectamine™ 2000 (Invitrogen™) and the expression of MGMT was examined by immunoblot. In parallel, the pcDNA-MGMT and pCDNA-transfected cells were used for radiation treatment and clonogenic assay.

### Statistical analyses

All data shown are the results of at least three independent experiments and are expressed as the mean ± standard deviation (SD). Student’s *t* tests (two-tailed) were used to analyse the significance of differences between groups. *P* < 0.05 was considered as significant.

## Additional Information

**How to cite this article**: Chen, S. *et al.* Endoplasmic reticulum protein 29 (ERp29) confers radioresistance through the DNA repair gene, O^6^-methylguanine DNA-methyltransferase, in breast cancer cells. *Sci. Rep.* **5**, 14723; doi: 10.1038/srep14723 (2015).

## Supplementary Material

Supplementary Information

## References

[b1] Ellgaard L. & Ruddock L. W. The human protein disulphide isomerase family: substrate interactions and functional properties. EMBO reports 6, 28–32 (2005). 15643448 10.1038/sj.embor.7400311 PMC1299221

[b2] Zhang D. & Richardson D. R. Endoplasmic reticulum protein 29 (ERp29): An emerging role in cancer. Int J Biochem Cell Biol 43, 33–36 (2011). 20920593 10.1016/j.biocel.2010.09.019

[b3] Rainey-Barger E. K., Mkrtchian S. & Tsai B. The C-terminal domain of ERp29 mediates polyomavirus binding, unfolding, and infection. J Virol 83, 1483–1491 (2009). 19019959 10.1128/JVI.02057-08 PMC2620899

[b4] Walczak C. P. & Tsai B. A PDI family network acts distinctly and coordinately with ERp29 to facilitate polyomavirus infection. J Virol 85, 2386–2396 (2011). 21159867 10.1128/JVI.01855-10 PMC3067762

[b5] Baryshev M., Sargsyan E. & Mkrtchian S. ERp29 is an essential endoplasmic reticulum factor regulating secretion of thyroglobulin. Biochemical and biophysical research communications 340, 617–624 (2006). 16380091 10.1016/j.bbrc.2005.12.052

[b6] Das S. *et al.* ERp29 restricts Connexin43 oligomerization in the endoplasmic reticulum. Molecular biology of the cell 20, 2593–2604 (2009). 19321666 10.1091/mbc.E08-07-0790 PMC2682600

[b7] Suaud L. *et al.* ERp29 regulates DeltaF508 and wild-type cystic fibrosis transmembrane conductance regulator (CFTR) trafficking to the plasma membrane in cystic fibrosis (CF) and non-CF epithelial cells. The Journal of biological chemistry 286, 21239–21253 (2011). 21525008 10.1074/jbc.M111.240267 PMC3122184

[b8] Reya T., Morrison S. J., Clarke M. F. & Weissman I. L. Stem cells, cancer, and cancer stem cells. Nature 414, 105–111 (2001). 11689955 10.1038/35102167

[b9] Bambang I. F., Lee Y. K., Richardson D. R. & Zhang D. Endoplasmic reticulum protein 29 regulates epithelial cell integrity during the mesenchymal-epithelial transition in breast cancer cells. Oncogene 32, 1240–1251 (2013). 22543584 10.1038/onc.2012.149

[b10] Farmaki E., Mkrtchian S., Papazian I., Papavassiliou A. G. & Kiaris H. ERp29 regulates response to doxorubicin by a PERK-mediated mechanism. Biochimica et biophysica acta 1813, 1165–1171 (2011). 21419175 10.1016/j.bbamcr.2011.03.003

[b11] Qi L. *et al.* Inhibiting ERp29 expression enhances radiosensitivity in human nasopharyngeal carcinoma cell lines. Medical oncology 29, 721–728 (2012). 21479953 10.1007/s12032-011-9929-5

[b12] Zhang D. & Putti T. C. Over-expression of ERp29 attenuates doxorubicin-induced cell apoptosis through up-regulation of Hsp27 in breast cancer cells. Experimental cell research 316, 3522–3531 (2010). 20833165 10.1016/j.yexcr.2010.08.014

[b13] Gao D. *et al.* ERp29 induces breast cancer cell growth arrest and survival through modulation of activation of p38 and upregulation of ER stress protein p58IPK. Laboratory investigation; a journal of technical methods and pathology 92, 200–213 (2012). 10.1038/labinvest.2011.163 22064321

[b14] Minotti G., Menna P., Salvatorelli E., Cairo G. & Gianni L. Anthracyclines: molecular advances and pharmacologic developments in antitumor activity and cardiotoxicity. Pharmacol Rev 56, 185–229 (2004). 15169927 10.1124/pr.56.2.6

[b15] Forrest R. A. *et al.* Activation of DNA damage response pathways as a consequence of anthracycline-DNA adduct formation. Biochem Pharmacol 83, 1602–1612 (2012). 22414726 10.1016/j.bcp.2012.02.026

[b16] Wood R. D., Mitchell M. & Lindahl T. Human DNA repair genes, 2005. Mutat Res 577, 275–283 (2005). 15922366 10.1016/j.mrfmmm.2005.03.007

[b17] Pegg A. E. Repair of O(6)-alkylguanine by alkyltransferases. Mutat Res 462, 83–100 (2000). 10767620 10.1016/s1383-5742(00)00017-x

[b18] Niture S. K., Doneanu C. E., Velu C. S., Bailey N. I. & Srivenugopal K. S. Proteomic analysis of human O6-methylguanine-DNA methyltransferase by affinity chromatography and tandem mass spectrometry. Biochemical and biophysical research communications 337, 1176–1184 (2005). 16226712 10.1016/j.bbrc.2005.09.177

[b19] Yan L., Donze J. R. & Liu L. Inactivated MGMT by O6-benzylguanine is associated with prolonged G2/M arrest in cancer cells treated with BCNU. Oncogene 24, 2175–2183 (2005). 15735757 10.1038/sj.onc.1208250

[b20] Gerson S. L. MGMT: its role in cancer aetiology and cancer therapeutics. Nat Rev Cancer 4, 296–307 (2004). 15057289 10.1038/nrc1319

[b21] Kaina B., Christmann M., Naumann S. & Roos W. P. MGMT: key node in the battle against genotoxicity, carcinogenicity and apoptosis induced by alkylating agents. DNA Repair (Amst) 6, 1079–1099 (2007). 17485253 10.1016/j.dnarep.2007.03.008

[b22] Bambang I. F. *et al.* Overexpression of endoplasmic reticulum protein 29 regulates mesenchymal-epithelial transition and suppresses xenograft tumor growth of invasive breast cancer cells. Laboratory investigation; a journal of technical methods and pathology 89, 1229–1242 (2009). 10.1038/labinvest.2009.87 19770839

[b23] Zmetakova I. *et al.* Evaluation of protein expression and DNA methylation profiles detected by pyrosequencing in invasive breast cancer. Neoplasma 60, 635–646 (2013). 23906298 10.4149/neo_2013_082

[b24] Keil K. P. *et al.* DNA methylation of E-cadherin is a priming mechanism for prostate development. Developmental biology 387, 142–153 (2014). 24503032 10.1016/j.ydbio.2014.01.020 PMC3976955

[b25] Harper J. W. & Elledge S. J. The DNA damage response: ten years after. Mol Cell 28, 739–745 (2007). 18082599 10.1016/j.molcel.2007.11.015

[b26] Rogakou E. P., Pilch D. R., Orr A. H., Ivanova V. S. & Bonner W. M. DNA double-stranded breaks induce histone H2AX phosphorylation on serine 139. J Biol Chem 273, 5858–5868 (1998). 9488723 10.1074/jbc.273.10.5858

[b27] Furdui C. M. Ionizing radiation: mechanisms and therapeutics. Antioxidants & redox signaling 21, 218–220 (2014). 24702164 10.1089/ars.2014.5935 PMC4060773

[b28] Jamieson D. & Boddy A. V. Pharmacogenetics of genes across the doxorubicin pathway. Expert opinion on drug metabolism & toxicology 7, 1201–1210 (2011). 21919804 10.1517/17425255.2011.610180

[b29] Okano M., Bell D. W., Haber D. A. & Li E. DNA methyltransferases Dnmt3a and Dnmt3b are essential for *de novo* methylation and mammalian development. Cell 99, 247–257 (1999). 10555141 10.1016/s0092-8674(00)81656-6

[b30] Varier R. A. & Timmers H. T. Histone lysine methylation and demethylation pathways in cancer. Biochimica et biophysica acta 1815, 75–89 (2011). 20951770 10.1016/j.bbcan.2010.10.002

[b31] Santos-Rosa H. *et al.* Active genes are tri-methylated at K4 of histone H3. Nature 419, 407–411 (2002). 12353038 10.1038/nature01080

[b32] Limp-Foster M. & Kelley M. R. DNA repair and gene therapy: implications for translational uses. Environmental and molecular mutagenesis 35, 71–81 (2000). 10712740 10.1002/(sici)1098-2280(2000)35:2<71::aid-em1>3.0.co;2-p

[b33] Pouliliou S. & Koukourakis M. I. Gamma histone 2AX (gamma-H2AX)as a predictive tool in radiation oncology. Biomarkers : biochemical indicators of exposure, response, and susceptibility to chemicals 19, 167–180 (2014). 10.3109/1354750X.2014.898099 24611829

[b34] Geric M., Gajski G. & Garaj-Vrhovac V. Gamma-H2AX as a biomarker for DNA double-strand breaks in ecotoxicology. Ecotoxicology and environmental safety 105, 13–21 (2014). 24780228 10.1016/j.ecoenv.2014.03.035

