# Supplementary material for: Endoplasmic reticulum protein 29 (ERp29) confers radioresistance through the DNA repair gene, O6-methylguanine DNA-methyltransferase, in breast cancer cells
Source: Sci Rep. 2015 Sep 30;5:14723. doi: 10.1038/srep14723 (PMC4588584; doi:10.1038/srep14723)

Endoplasmic reticulum protein 29 (ERp29) confers radioresistance through the DNA repair gene, O^6-methylguanine DNA-methyltransferase, in breast cancer cells

Shaohua Chen, Yu Zhang, Daohai Zhang

Suppl. Figure 1. Knockdown efficiency of target genes using the Trilencer-27 human siRNA duplex. Cells at 60-70 % confluence were transfected with the indicated siRNA and control siRNA (final concentration, 50 nM). After 48 hours, cells were harvested and the expression of target genes was examined by Western blot. The level of protein expression was normalised to internal control (β-actin). Knockdown efficiency was evaluated by comparing to the cells treated with control siRNA. Data represent a mean of three independent experiments.

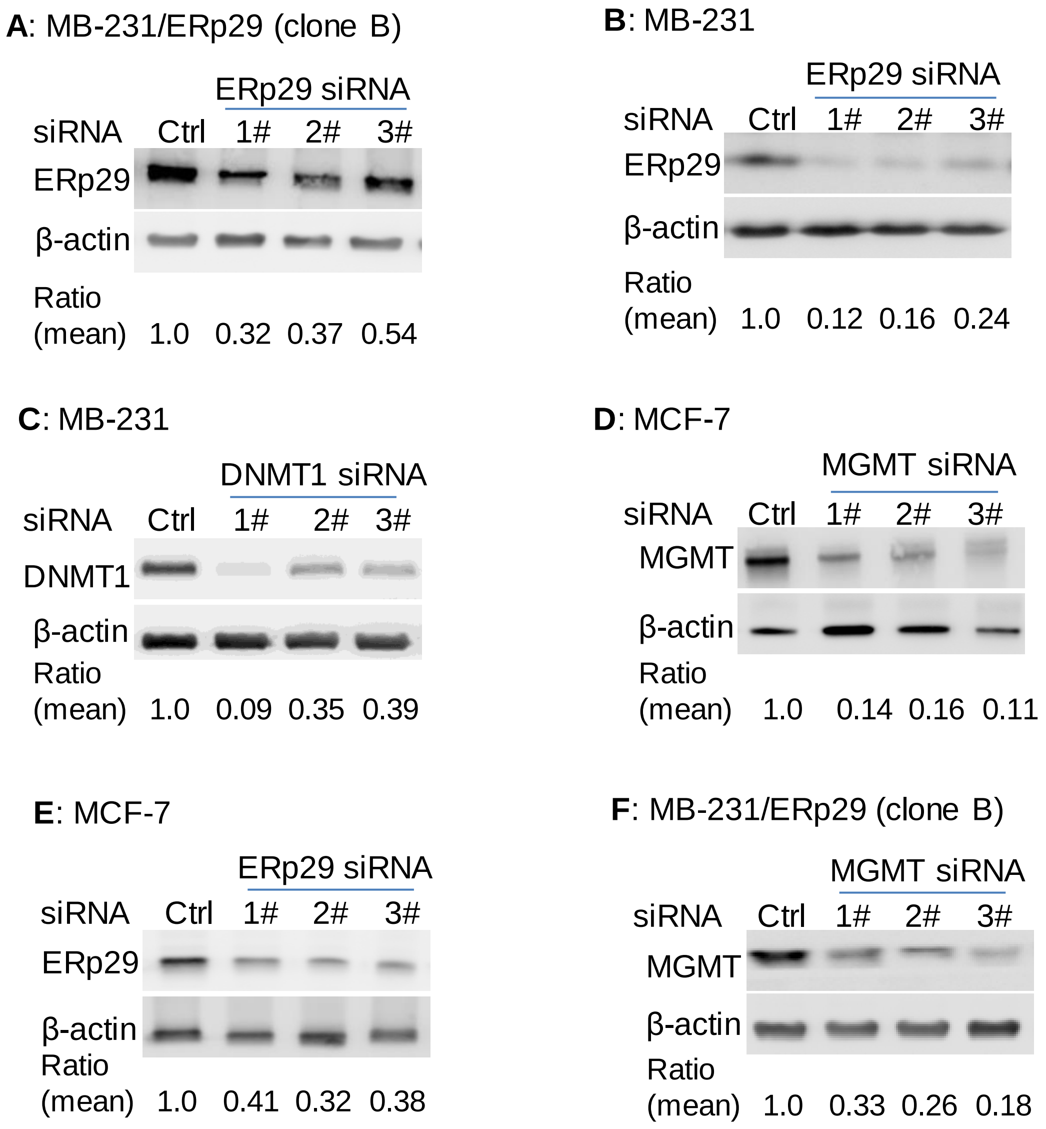

Supplement: Supplementary Information [file srep14723-s1.pdf]
